# Perspectives of French community-dwelling older adults on the use of digital services: a qualitative study based on interviews

**DOI:** 10.1186/s12877-026-07511-w

**Published:** 2026-05-09

**Authors:** J. Cattoni, A. S. Rigaud, S. Dacunha, E. Perrot, M. Chetouani, M. Pino

**Affiliations:** 1 Service Gériatrie 1&2, Centre Mémoire de Ressources et Recherches Ile-de-France-Broca, AP-HP, Hôpital Broca, Paris, France; 2 Broca Living Lab, Paris, France; 3 INSERM Optimisation Thérapeutique en Pharmacologie OTEN U1144, Université Paris Cité, Paris, France; 4 ISIR, CNRS UMR7222, Inserm ERL U1150, Sorbonne Université, Paris, France

**Keywords:** Older adults, Digital services, Accessibility, Digital exclusion

## Abstract

**Background:**

Everyday services are increasingly being digitalised, while the proportion of older adults continues to grow. Ensuring that digital services are accessible to this population is essential to prevent digital exclusion and support autonomy. This study aimed to explore older adults’ perspectives, experiences, and needs regarding digital services, identify perceived barriers to accessibility, and examine associated ethical and societal concerns.

**Methods:**

This qualitative descriptive study recruited 48 French community-dwelling older adults in France (28 women, 20 men; mean age = 76.06 years, SD = 9.21; 42 urban, 6 rural). Semi-structured interviews were conducted using an 18-item guide developed from a review of the literature on digital accessibility. The study design and reporting were informed by the COREQ (Consolidated Criteria for Reporting Qualitative Research) checklist. Interviews were audio-recorded, transcribed verbatim, pseudonymised, and analysed using inductive thematic analysis. Coding was performed independently by two researchers, and discrepancies were resolved through discussion.

**Results:**

All participants reported using at least one digital technology, primarily for communication, information-seeking, administrative procedures, entertainment, and, more rarely, for professional or obligatory tasks. Participants described both advantages (e.g., convenience, ergonomic interfaces) and difficulties (e.g., lack of support, interface complexity, technical failures), leading to diverse coping strategies such as seeking help, self-learning, delegation, or avoiding digital tools when possible. Feelings toward digitalisation were ambivalent, combining perceived benefits with frustration, concerns about data security, and reflections on the impact of digitalisation on autonomy and human relationships. Participants also identified barriers to accessibility (e.g., lack of digital literacy, financial constraints, poor design, insufficient support) and proposed concrete solutions, emphasising the importance of accessible design, non-digital alternatives, adapted training, and human assistance.

**Conclusions:**

Digitalisation offers opportunities for older adults but also raises accessibility, ethical, and societal challenges. While many participants recognised the usefulness of digital services, they also highlighted risks related to autonomy, perceived choice, and reduced human contact. These findings underscore the need for digital services that are inclusive, intuitive, and supported by continuous human assistance to avoid reinforcing existing inequalities.

**Supplementary Information:**

The online version contains supplementary material available at 10.1186/s12877-026-07511-w.

## Background

In 2024, according to Eurostat-EU, the proportion of the population aged 65 and over in Europe stood at 21.6%, an increase of 5.2% points in twenty years. At the same time, in recent years we have seen the digitalisation of many services, combined with the rapid spread of new technologies in most areas of society (work, education, healthcare, communication, entertainment) [1]. These technological advances provide considerable assistance to human beings in many areas. However, they also lead to the exclusion of certain populations by creating gaps between those who can use these new services and those who cannot access them: this is the concept of the digital divide [2]. This concept, developed to explain differences in technology use among different population groups, refers to inequalities in access to digital services and the skills needed to use them, linked in particular to geographical, socioeconomic, educational, generational, and health factors [2]. Older adults are particularly affected by the digital divide, as they face multiple barriers to accessing and using technology (e.g. usability and accessibility issues, lack of confidence/experience, costs, etc.) [3–5]. Older adults therefore keep their distance from this digital transition and are slower to adopt new technologies than younger age groups [3, 6–8], raising questions about their inclusion in the digital society [9].

A key question in the current context is how to take into account the abilities, needs and preferences of older adults in the design and use of digital systems [10]. Consideration of sensory, cognitive, socio-demographic, psychosocial and human factors is essential here, as how people interact with technology and their confidence in their ability to use it will affect its effective use and long-term adoption [11]. However, the heterogeneity within the older adult group must also be taken into account (e.g., age, abilities, health status, technological experience). Consequently, various accessibility needs are observed in this age group. To create accessible digital services, it is important to have a comprehensive understanding of the barriers older adults in their diversity may face when using each element of the service, based on their skills, abilities and preferences [12]. In this context, accessibility is a crucial aspect that can be understood in two ways: in terms of perceptual, cognitive and psychosocial dimensions (visual, auditory, physical manipulation, cognitive abilities, self-efficacy, etc.) and tangible access to technologies, which may be more or less available to individuals depending on their social and technological environment, personal situation, needs and financial resources [13, 14].

### Technology and older adults: contrasting views

#### Specific contributions

Today, society and older adults themselves tend to favor, whenever possible, home care and support for independence as much as their situation allows [15–17]. To this end, the use of new technologies and digital services by older adults offers a number of advantages and benefits. For example, online communication services, telemedicine and paperless administrative procedures could make it easier for older adults to access essential resources when they are able to use these services [10]. These tools can reinforce a sense of independence and improve quality of life, particularly when they enable people to stay in touch with family, manage their health or simplify certain daily tasks [7, 11]. For some older adults, technology can also represent a form of access to progress, understood as the ability to remain connected to evolving services, information, and social practices [18].

The perceived benefits are not limited to functional autonomy. Several studies also highlight the positive impacts in terms of well-being, cognitive stimulation and reduced social isolation through the use of communication technologies and social networks [19–21]. For example, Internet use among older adults has been associated with a reduction in depressive symptoms [22], a stronger sense of community belonging [23] and greater life satisfaction [24, 25].

Thus, although the adoption of technologies is slower among older adults, these tools have significant potential to support their independence and quality of life [12, 26].

#### Attitudes, perceptions and issues specific to older adults

Older adults’ attitudes towards technology can be however ambivalent and marked by contrasting social representations. While some perceive digital tools as an opportunity to stay connected, informed and independent, others consider them complex, inaccessible or even useless in their daily lives [7, 27]. This heterogeneity reflects not only generational and socio-economic differences, but also varied life trajectories in terms of exposure to technology. It is also evident within the older adult group itself: younger seniors, who are often familiar with digital technology from their professional careers, adopt these tools more easily than older adults, who were exposed to digitalisation after retirement [3, 28–30]. Studies show that, in general, digital usage drops significantly around the age of 75: people aged 65–74 are much more connected than those aged 75–79, or even those aged 80 and over [31, 32]. This diversity requires us to move beyond a uniform vision and design adaptive, inclusive and customisable devices [10].

Furthermore, attitudes towards technology among older adults can be influenced by previous negative experiences, a lack of familiarity, or increased technological anxiety with age [1, 33]. These psychological barriers play a role in computer use and affect perceived competence, hindering learning and adoption of digital tools [34, 35]. Barriers to technology adoption also include insufficient training [36], cognitive, sensory, perceptual and motor decline that can interfere with the ability to interact with these devices [1, 37–39], and the rapid pace of technological change [40]. In addition, significant concerns about security [41–43] and/or trust in technology [26] can discourage older adults from using such tools. Finally, socio-economic and educational inequalities play a central role. People with low levels of education, income or living in rural areas are more affected by the digital divide [3, 5]. These multiple barriers contribute to accentuating the digital exclusion of some older adults, despite the documented benefits in terms of well-being and social connection when technologies are effectively adopted.

The relationship between older adults and technology is more nuanced than the common stereotype that they are simply fearful or reluctant to use digital tools. As shown by Mitzner et al. [33] many older adults express positive attitudes and recognise the benefits of digital technologies in their everyday lives. Recent studies similarly report that most older adults now use at least one digital device (smartphone, computer, tablet) for communication or information [6, 44, 45]. Nevertheless, the scope and nature of their digital practices differ from those of younger populations. Older adults’ use tends to be selective and utilitarian, concentrating on communication, information seeking and essential services, while younger age groups generally adopt a broader range of uses, including leisure activities, content creation and social media engagement [3, 29].

### Digital accessibility for older adults: progress, limitations, and current challenges

In recent decades, numerous efforts have been made to make technologies more accessible to older adults, and several studies show that the adoption of digital services depends heavily on perceived usefulness and ease of use [10, 44, 46, 47], two criteria whose improvement has already been the subject of research aimed at identifying levers for action. Despite these advances, all of these technologies still have a low adoption rate among older adults [48], which has led to attempts to formulate guidelines to improve their accessibility: provide useful information, facilitate communication with loved ones [48], design more user-friendly, less complex tools [49] and adapted to age-related perceptual, cognitive, and motor limitations [49, 50], ensure system robustness [51], offer training and personalized support [26, 41], and use clear vocabulary overall [49, 52]. Finally, including older adults in the design and evaluation processes promotes more robust, relevant and accepted technologies. A participatory approach, involving multiple stakeholders (users, carers, professionals, designers), makes it possible to develop digital services that are truly adapted to the needs and preferences of this population [19, 53].

However, even if these recommendations help make technologies more accessible, the needs, capabilities, and expectations of older adults are constantly evolving in parallel with rapid digital transformations. The study presented here is therefore of crucial importance in providing an up-to-date perspective on their needs and views on digitalisation, and in better understanding the conditions that will enable sustainable improvements in the accessibility of technologies. In particular, this study contributes by capturing older adults’ own accounts of digitalisation at a moment when digital services are increasingly mandatory rather than optional, thereby shedding light on how perceived choice, dependence and accessibility are experienced in everyday contexts. By focusing on lived experiences and perceptions rather than adoption rates alone, it provides insights that complement existing guideline-based and technology-centred approaches. Furthermore, recent studies show that cultural and national contexts, through cultural capital or conditions of access and adaptation, strongly influence how older adults perceive, use, and accept technologies [54, 55]. It is therefore essential to survey older French people in order to understand their specific points of view on digitalisation.

Implementing an accessible digital service that caters to a wide range of users, and specifically older adults who may find it more difficult to adopt these tools, requires a thorough understanding of the barriers to interaction that people may face when using each component of the application, depending on their skills, abilities and preferences. The objectives of this study are to gain an in-depth understanding of (a) how French older adults describe their everyday experiences with digital services, including their patterns of use, adaptive strategies, and attitudes toward these technologies; (b) older adults’ perceptions of barriers to digital accessibility and possible solutions; and (c) older adults’ views on ethical and societal challenges related to digital services, accessibility, digital inclusion and social inclusion. Finally, this study will aim to inform recommendations for promoting the accessibility of digital services, including current and local perspectives.

## Methods

The methodological approach adhered to the 32 criteria of the COREQ (Consolidated Criteria for Reporting Qualitative Research) framework [56] ensuring transparency and rigor throughout the research process. Compliance with the COREQ criteria is described in detail below (Annex 1).

### Study design

This qualitative study was based on semi-structured interviews with French older adults aged 60 or over, living in France. It was conducted between May and November 2023 by JC (Female, PhD student in cognitive science). The interviewer had prior experience conducting qualitative studies and had received formal training in semi-structured interviewing techniques.

### Participants

Participants were recruited through senior citizens’ associations in Ile-de-France and Loire-Atlantique, mainly via email but also with flyers. A total of 48 older adults participated in the interviews. Nobody dropped out from the study. The socio-demographic data of the participants are shown in Table 1. A complete description of the socio-demographic data with the corresponding participant code is provided in Annex 2.

**Table 1:** Socio-demographic data of participants

Variables	Modalities	Results
Number of participants	-	*n* = 48
Sex	Male	*n* = 20 (41,7%)
	Female	*n* = 28 (58,3%)
Age	-	Mean = 76,06 SD = 9,21 Min = 60 Max = 96
Years of schooling	5 (end of elementary school), 9 (end of middle school) 12 (high school graduation and beyond)	Mean = 13,9 SD = 3,5 Min = 5 Max = 20
Living environment	Urban	*n* = 42 (87,5%)
	Rural	*n* = 6 (12,5%)

### Material

#### Interview guide

An interview guide of 18 questions was developed and is detailed in Annex 3. To ensure question validity for the interviews, the following steps were taken. Firstly, two researchers (JC and SD - MSc in Psychology) wrote the questions based on prior research on digital accessibility and technology use among older adults [57–59]. Secondly, the interview guide was reviewed by three additional researchers (MP - Dr in Psychology, ASR - MD in Geriatrics, and MC - Professor of Human-Machine Interaction) with expertise in ageing and digital technologies to assess the relevance, clarity and comprehensiveness of the questions in relation to the study objectives. Thirdly, a small set of pilot interviews (*n* = 7) was conducted with older adults to test the guide in practice and identify potential ambiguities or difficulties in understanding. Minor adjustments were subsequently made to simplify wording and improve the clarity of some questions. The dimensions addressed in the interview grid and some examples of related questions are presented in Table 2.

**Table 2:** Areas of study and examples of questions

Themes	Examples
How do older adults approach the use of digital services?	• What technologies do you use in your daily life? • Do you find these digital services easy to use?
Older adults’ perception of barriers to digital accessibility and possible solutions	• Could this dynamic of digitalisation lead to the exclusion of certain groups? • What suggestions would you have for improving the accessibility of digital services for older adults?
Older adults’ views on ethical and social challenges relating to digital services, accessibility, digital inclusion, and social inclusion	• Are you concerned about the confidentiality of your personal information when using digital services? • What is your opinion/view on the increasing digitalisation of services?

### Procedure

Participants were invited to take part in semi-structured interviews designed to explore the relationship between older adults living in France and digital services, as well as the obstacles they may encounter in using them. Interviews were conducted either in person at a research laboratory within a french geriatric facility or via videoconference for a few of them. We established dedicated schedules that allocated specific time slots for volunteer participants. Only the study participants and the researcher were present during the interview and the researcher had no prior relationship with the participants before the study began.

Prior to participation, each individual received an information sheet describing the objectives of the project, either in advance (for videoconference interviews) or on the day of the interview (for face-to-face interviews). Written informed consent was obtained using a consent form provided in duplicate. Participants also completed a short questionnaire collecting socio-demographic information (age, gender, educational level, living environment, socio-professional category) and two questions regarding their frequency of use and level of comfort with digital services (“How often do you use technology?” and “How comfortable do you feel using technology?”) in order to obtain an initial overview of their habits and self-perceived ease with digital tools.

The interviews were then carried out following the themes of the interview guide. No field notes were taken during the interviews. Each participant was interviewed once, and transcripts were not returned for feedback or revision. Data collection continued until the research team considered that thematic saturation had been reached, defined as the point during the coding process at which additional interviews no longer generated new themes or codes relevant to the research questions (e.g. Table 3). All data were anonymised and treated confidentially in accordance with ethical research standards. This study was reviewed and approved by the Research Ethics Committee of CEGIF, under reference 52,023 in 2023.

### Data analysis

The interviews were recorded with a dictaphone, and transcribed. The average interview duration was 24.5 min (SD 10.6 min). The content of the interviews was analyzed with MAXQDA (version 2020) software using an inductive thematic analysis approach, following the principles of Braun and Clarke [60]. This approach involves reviewing the interviews several times to fully understand their content and to identify and organize the topics covered in order to improve the results. Following the initial phase of encoding the interviews, the codes were grouped into themes, then analyzed and classified according to the three main objectives of the study. Finally, the themes were labeled, with examples given to explain each one. To assess consistency between evaluators in the analysis of the interviews, we adopted a systematic method involving two researchers (JC, EP - MSc in cognitive sciences). Both researchers were experienced in the field of technologies accessible to older adults. Each researcher independently performed an inductive thematic analysis and then, they compared the content of the two codings and assessed their level of agreement. In the event of disagreement, the two researchers discussed and eventually redefined the codes together. A third researcher (MP) then reviewed the codes to validate the thematic map. This process ensured a consistent and reliable coding framework, thereby enhancing the validity of our study’s findings. All interview data was pseudonymized. Findings were not returned to participants for feedback. As the interviews were semi-structured, any ambiguities or unclear responses were clarified during the interview through follow-up questions and discussion with the participants.

An exploratory quantitative analysis was carried out using R software (version 4.5.2), focusing on three dimensions: sociodemographic data, self-assessed level of comfort with technology, and number of mentions of the different subcodes of the thematic map. For sociodemographic characteristics (age, gender, socio-educational level, living environment, socio-professional category), descriptive statistics (mean, standard deviation, minimum and maximum) were first calculated. The relationships between technological comfort and the sociodemographic data were analyzed using Spearman’s correlations, to determine whether individual differences were associated with self-assessed comfort with technology. We also examined differences in the frequency of mention of the different subcodes between participants under the age of 75 and those aged 75 years and over. For each subcode, differences in proportions between the two groups were assessed using chi-square tests of independence applied to 2 × 2 contingency tables, based on binary mention data (mentioned vs. not mentioned).

## Results

**Fig. 1:**
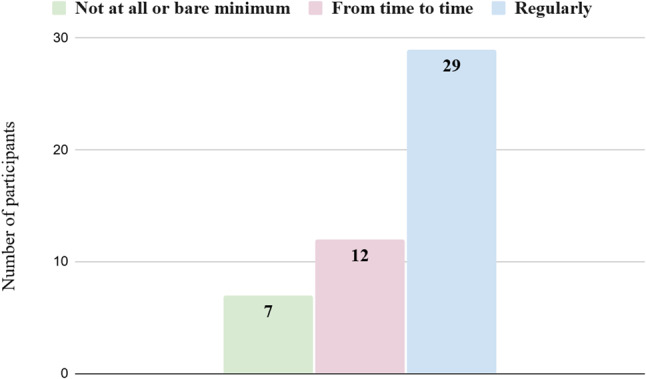
Distribution of participants according to frequency of technology use

**Fig. 2:**
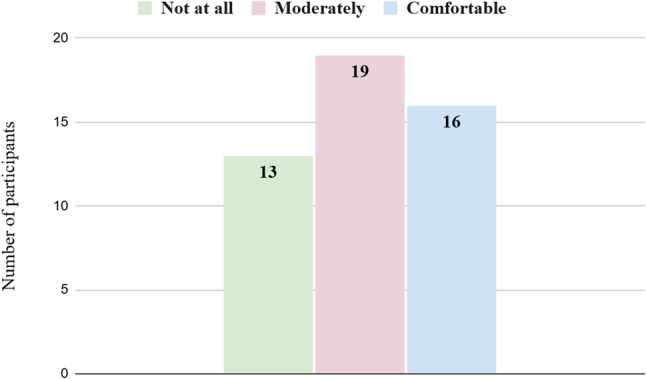
Distribution of participants according to self-reported comfort with technology

The distribution of participants’ responses regarding frequency of technology use and self-reported ease with technology is presented in Figs. 1 and 2.

The qualitative analysis of the interviews identified three main themes aligned with the study objectives: (a) older adults’ descriptions of their everyday experiences with digital services, including their patterns of use, adaptive strategies, and attitudes toward these technologies; (b) older adults’ perceptions of barriers to digital accessibility and potential solutions; and (c) their views on ethical and societal challenges related to digital services, accessibility, digital inclusion, and social participation. Across these three themes, a total of 12 codes and 55 subcodes were identified, as detailed in Annex 4. Each subcode is presented with a definition and an illustrative verbatim extract. Table 3 reports the number of participants (out of 48) who mentioned each subcode.

**Table 3:** Frequency of subcode mentions across interviews (*n* = 48)

THEME	CODE	SUBCODE	Occurrence of subcodes across interviews (*n* = 48)
(1) How older adults describe their everyday experiences with digital services, including their patterns of use, adaptive strategies, and attitudes toward these technologies	Reasons for use	Work	10
		By obligation	22
		Communication	35
		Informational	30
		Administratives formalities	25
		Entertainment	28
	Facilitating factors	Ease of use	30
		Being used to	23
		Good ergonomics	10
	Limiting factors	Difficulty of use	33
		No being used to	34
		Technical failures	17
		Lack of user support and documentation	22
		Poor ergonomics	34
	Coping strategies	Human assistance	45
		Delegation of the task	14
		Dependency	20
		Preference for human service	18
		Seek for non digital alternative	25
		Learning/Adaptation	35
	Attitudes and experiences of digital technology	Feeling of competence	26
		Generational gap	39
		Lack of concern	16
		Feeling at ease	27
		Reluctance toward technology	33
		Abandonment/Waiver	27
		Irritation/Frustration	33
		Determination to use technology	32
(2) Older adults’ perceptions of barriers to digital accessibility and possible solutions	Barriers to accessibility	Digital literacy	29
		Lack of age-inclusive design	40
		Socio-educational level	11
		Geographical	12
		Affordability	13
		Feeling of exclusion	39
	Suggestions to improve accessibility	Human support	31
		Training	21
		Inclusive design	29
		Having the motivation to adapt	22
(3) Older adults’ views on ethical and societal challenges related to digital services, accessibility, digital inclusion and social inclusion	Positives aspects	Opportunities/Simplification	34
		Improvement of sociabilisation	17
		Enjoyment of technology use	34
	Negative aspects	Future loss of control	13
		Risk of malfunction	17
		Loss of human connection	28
		Digitisation perceived as detrimental	31
		Risk of human replacement	14
	Perceived choice in digital use	Existing choice	28
		Ephemeral choice	12
		Imposed use	40
	Justifications for widespread digitisation	Perceived as part of progress	33
		More ecological	8
		Economic gain	17
		Time saving	32
	Perceptions of digital security	Confidentiality risks	43
		Low concern about digital security	21

### Theme 1: digital approaches and experiences

#### Technologies used and motivations for use

All respondents reported using at least one form of digital technology in their daily lives, including smartphones (100%), computers (69%), tablets (46%), and, less frequently, connected devices (4%). In addition, 54% of older adults indicated that they used self-service terminals in contexts such as train stations and airports, restaurants, supermarkets (self-checkout), cinemas, or for cash withdrawal at automated teller machines. This widespread exposure to digital technologies suggests a high level of everyday familiarity. However, the frequency of use and perceived proficiency varied considerably across individuals.

Participants reported multiple reasons for using digital technologies, reflecting both functional needs, such as completing online administrative procedures, accessing information, and communicating, and more personal motivations, including entertainment and maintaining social ties. Communication emerged as the most commonly cited reason for use, reported by 73% of respondents. In this context, digital tools, mainly smartphones and messaging applications, were described as essential for maintaining social relationships: *“I send emails to keep in touch with people who are far away*, etc.,* or for quick communication.” (P023AN)*, particularly with their loved ones: *”I mainly use it to contact family and friends” (P007AN)*. These practices were perceived as helping to reduce feelings of isolation and were often associated with a positive view of digital technologies as facilitators of human connection.

Informational use emerged as the second most common purpose for digital technology use, reported by 63% of respondents. Participants described using the Internet to stay informed and to learn, as illustrated by one interviewee: *“So in everyday life*,* I use mainly to keep myself informed*,* to educate myself…” (P034AN)*. Entertainment was also frequently mentioned (58%), including online games and watching films or series. For example, one participant noted, *“I play Scrabble because I enjoy it*,* with my friends who are on the other side of the world right now” (P020AN).* These practices were often associated with enjoyment and leisure, suggesting that digital technologies extend beyond purely functional purposes.

By contrast, some older adults reported using digital tools primarily out of necessity (46%), particularly when no alternative was available. As one participant explained, *“I use it*,* but only when there’s no other option.” (P013AN)*. This was especially evident for administrative tasks, cited by 52% of respondents as a regular use, including making medical appointments via platforms such as Doctolib, managing finances, or filing tax returns. One participant remarked, *“Oh yeah*,* I do my banking online. I’ve gone paperless for everything*,* bills*,* whether it’s at my holiday home or even here*,* all my subscriptions*,* well*,* all my bills are paperless.” (P043AN)*.

Finally, 21% of respondents mentioned work-related use of digital technologies, mainly prior to retirement: *“So it helps me work*,* it helps me access the things I work with*,* whether it’s email*,* writing*,* word processing*,* or document software.” (P030AN)*. This suggests that previous professional exposure, and in some cases ongoing professional activity among the youngest participants, may shape current familiarity with and attitudes toward digital tools.

#### Facilitating and limiting factors in the use of technology

The interviews reveal considerable heterogeneity in terms of digital literacy and proficiency. Several participants mention familiarity acquired over time, often linked to being used to using technology (48%), as one participant noted: *“That’s it*,* I know it now*,* it’s a learning process” (P021AN)*. Others emphasized the role of regular practice in building confidence, for example, *“The technologies I use most of the time work well for me.” (P044AN)*. In addition, some participants highlighted the importance of device usability, with 21% referring to the good ergonomics of certain tools: *“Generally*,* it’s quite ergonomic. You have the photos*,* you just have to click on them” (P043AN)*.

Conversely, many respondents reported persistent difficulties in using digital technologies, often linked to a perceived generational gap: *“I lack the technical vocabulary that young people have immediately*,* with really precise words” (P006AN); “It’s not our generation*,* we weren’t born into it” (P001AN)*. Several participants described difficulties engaging with unfamiliar technologies because of their novelty (71%) and a sense of losing their bearings in constantly evolving digital environments: *“I find it hard to learn new things*,* and this is the archetype of new things to learn*,* eh” (P032AN)*.

Other obstacles included technical failures (35%), with participants pointing out that technologies do not always function as expected, for example, *“there are a lot of bugs” (P011AN)*. Many also emphasized the complexity of tools and interfaces (71%), particularly due to dense information displays and the cognitive effort required to understand how to proceed: *“I can’t use it immediately given my background*,* so I think to myself*,* this device is poorly designed.” (P014AN)*. Finally, a lack of accessible information and support when adopting new technologies was frequently mentioned (46%): *“they put you in front of a machine and basically say: “well*,* figure it out for yourself!”” (P020AN)*.

#### Attitudes towards digitalisation

The interviews highlight a wide range of attitudes and experiences in relation to digital technology. Some participants described an overall sense of ease or positive attitudes (56%), expressing confidence in their ability to use digital services without difficulty (*“So there you go*,* it’s my domain [digital tools*,* right?” (P016AN)*), and sometimes even admiration for what digital technologies can offer: *“I’m amazed by the increasing user-friendliness*,* the performance of the devices*,* their responsiveness” (P014AN)*, or *“It’s absolutely wonderful*,* that’s for sure. For everything administrative*,* it’s wonderful. I think it’s great” (P022AN)*.

Conversely, others questioned their own skills (54%), expressing doubt, stress, or fear about their ability to act appropriately or efficiently: *“I panic as soon as I don’t know something*,* as soon as I tell myself I won’t be able to do it because I have so many doubts. It’s terrible. I get stressed.” (P003AN)*, or *“The fear of yes*,* (…)*,* the fear of not succeeding and then wasting other people’s time. The shame*,* yes*,* that’s a bit of it.” (P008AN)*. These feelings were often associated with the perception that age itself (81%) constitutes a barrier to learning or adaptation: *“How can I put it? The brain doesn’t work the same way in someone of a certain age as it does in someone of middle age or a child” (P002AN)*, or *“I can see that children get straight to it because they have that mental agility*,* whereas we were more Cartesian*,* pencil and paper…” (P006AN)*.

Between these two extremes, many participants adopted more nuanced positions, characterized either by reluctance toward digital services (69%), as illustrated by one participant who noted that *“there was a bit of stress at the beginning*,* but then it all worked out” (P026AN)*, or by a lack of concern (33%), reflecting the view that digital technology does not represent a priority in their daily lives. As one respondent remarked, *“And anyway*,* that’s not my main concern*,* eh*,* I sleep well at night*,* that’s for sure.” (P007AN)*.

For some, difficulties using digital services triggered stronger emotional reactions, ranging from frustration to irritation (69%), particularly in situations perceived as obstructive or incomprehensible, when systems failed to function as expected (*“but I get frustrated very quickly*,* let’s say. It should work” (P022AN)*) or when they were unable to achieve their goals (*“because when I try and it doesn’t work*,* you know*,* it makes me angry*,* it makes you face the fact that your brain doesn’t understand” (P024AN)*). These emotions sometimes led to attitudes of withdrawal or abandonment (56%): *“And then I often give up because it took me too long to find the answer*,* so I don’t really know how to get to the point.” (P042AN)*, or *“So I prefer not to do it*,* it’s not good” (P020AN)*.

However, others reacted in the opposite way, showing strong determination to persist (67%) through repeated attempts and a willingness to persevere. As one participant explained, *“So*,* I hang on and overcome my indifference in order to truly engage with the issue.” (P035AN)*. Another insisted, *“No*,* I’m not giving up*,* I’m trying*,* I’m trying everything.” (P006AN)*. This determination was echoed by those who emphasized active problem-solving: *“but I try hard to find solutions when I have problems” (P009AN)*.

#### Digital coping strategies adopted

Depending on their level of comfort with technology, older adults adopted a range of coping strategies to continue using digital tools despite the obstacles they encountered. These strategies reflected a desire to persevere and figure things out for themselves but also often a feeling that they had no other choice.

Seeking human assistance emerged as the most commonly reported strategy (94%). Many participants relied on family members or friends for support, either by asking for guidance, as illustrated by one respondent who said, *“But if it doesn’t work*,* I’ll go to a phone shop and tell them [the employees] that I don’t understand*,* and that it’s not working [the technology]” (P044AN)*, or by delegating tasks entirely to someone perceived as more competent: *“I do what I can*,* and then I leave the rest to people who are more qualified than me” (P017AN)*. However, this reliance on others was often described as ambivalent. While helpful, it could also generate feelings of dependence (42%), as expressed by participants who noted, *“I would have been lost without my sister” (P032AN)*, or *“You’re always dependent*,* forced to ask your children or grandchildren*,* which can be embarrassing” (P033AN)*. Some also feared burdening others, for example, *“I have nice neighbours*,* but I don’t want to bother them all the time” (P012AN)*.

Some participants also reported using digital forms of assistance, such as tutorials, chatbots, or voice-activated assistants (48%), but expressed limited trust in these tools:*“No*,* [chatbots] aren’t very convincing*,* not always*,* sometimes yes*,* sometimes no. " (P019AN)*), particularly because they felt these systems failed to provide answers that adequately addressed their specific questions: *“Yes*,* I have used a chatbot*,* for example*,* I don’t know*,* on insurance websites. It was an extremely interesting experience because it’s a bit like a snake biting its own tail*,* you go round in circles. Artificial intelligence still has a long way to go*,* doesn’t it? Because I’ve never got a satisfactory answer that way. " (P010AN)*.

Other participants described strategies of delegation or task sharing within the household (29%), for instance, *“My husband takes care of the taxes” (P024AN)*. Some preferred to bypass digital technologies altogether by favoring non-digital alternatives (52%), such as *“I still do my accounts the old-fashioned way*,* on paper and everything” (P023AN)*, often accompanied by a preference for face-to-face human services (38%): *“I prefer to ask someone for information” (P015AN)*, or *“Oh no*,* I queue up*,* I go to someone*,* it’s the same for paying at the supermarket. I hate self-service checkouts” (P044AN)*. Finally, a substantial proportion of respondents (73%) emphasized independent learning and gradual adaptation as key strategies. Participants described actively searching for solutions and learning through trial and error, as one explained: *“I do some research. YouTube is great*,* there are explanations. Well*,* for my photos*,* I managed by finding out how to do it” (P003AN)*. Thus, despite these constraints, most participants did not adopt a stance of total rejection toward digital technologies. Instead, they sought to adapt by drawing on their own abilities, social resources, and personal preferences. However, this process of adaptation, often framed as an individual effort, did not always reflect a genuine choice, but rather a response to limited alternatives.

### Theme 2 : perceptions of barriers to accessibility and possible solutions

#### Perceived barriers to digital accessibility

The interviews highlighted a set of structural and situational barriers that shape digital accessibility and condition opportunities for digital inclusion among older adults. Participants first emphasized age-related difficulties as a major barrier (83%), noting that digitalization is often not well aligned with the needs of older users. As one participant observed, *“It is developing very quickly without taking into account the age of some people” (P002AN)*, which was perceived as increasing the risk of exclusion. This lack of consideration was linked both to sensory, cognitive and functional limitations (e.g., vision, hearing, dexterity, slower information processing, etc): *“No [older adults are not considered in the design]*,* being older means seeing less well*,* understanding more slowly*,* perhaps having slightly less dexterous fingers*,* not hearing well” (P035AN)*, and to generational factors among those who had not been exposed to digital technologies earlier in life: *“It was so sudden and it evolved so quickly that the older generation*,* the people who didn’t get involved and who refused*,* saying to themselves*,* ‘well*,* we can live without it*,*’ they’re overwhelmed” (P043AN)*.

A second major barrier concerned limited digital literacy (60%), with participants stressing that some individuals lack even basic skills, for example, *“Some people don’t even know how to start a computer” (P025AN)*.

Access to digital infrastructure was also frequently mentioned (25%), particularly difficulties related to geographical location and unequal network coverage: *“I think there really are areas that don’t have any internet access at all” (P010AN)*. This was accompanied by concerns about territorial inequalities, as one respondent stated, *“We need to work upstream to ensure that large urban areas are not the only ones to benefit*,* when there is a lack of coverage in rural areas” (P002AN)*.

The financial cost of digital access emerged as another important barrier (27%), encompassing the price of devices, subscriptions, and renewals: *“We need to help people go digital because it’s extremely expensive for a family budget. Some families spend*,* let’s say*,* 20% of their budget on digital technology” (P038AN)*. Others noted that not everyone can afford adequate equipment: *“There are people who already don’t have the means to afford proper equipment” (P023AN)*.

Finally, socio-educational level was identified as a barrier by 23% of participants, who felt that limited literacy skills could hinder understanding of online interfaces and procedures: *“To connect*,* you have to know how to read and write” (P003AN)*. Language barriers were also mentioned, given the prevalence of English in digital environments: *“People who can’t read well don’t practise their English. Because a lot of things are in English. I think that’s a barrier” (P004AN)*.

Taken together, these barriers, whether technical, economic, geographical, or skill-related, contributed to a widespread feeling of exclusion or distance from essential services among participants, reported by 81%. As one respondent expressed, *“I think the gap is going to widen*,* and I don’t know how many people are going to manage” (P016AN)*, while another noted, *“The whole IT thing excludes people who can’t use it*,* and that can be all kinds of people who can’t use it” (P030AN)*.

#### Solutions envisaged to improve digital accessibility

In response to this experience of inaccessibility, participants also proposed several concrete ideas to improve current conditions and to support more equitable forms of digital inclusion. Training emerged as the most frequently mentioned solution (44%), with participants emphasizing the importance of trainers who are both pedagogically skilled and attuned to the needs of older adults. As one respondent noted, *“It has to be someone who has teaching skills suited to the target population*,* in order to provide clear and practical understanding for these individuals” (P002AN)*. Training was envisioned in various formats, including group workshops, *“We should have workshops*,* we need to develop workshops for us where we can go when we need help” (P003AN)*, individualized support, and local community initiatives, such as intergenerational programs or services organized by municipalities: *“You could even consider some kind of [training] service*,* free*,* with young people who will train older adults” (P024AN)*, and *“First of all*,* [training] services at the town hall*,* because that’s where people go*,* the little old people” (P031AN)*.

Participants also stressed the need to improve design and usability (60%), calling for interfaces and manuals that are more user-friendly, consistent, and better adapted to older users. One participant suggested, *“Perhaps something more progressive*,* perhaps a little more detailed so that you can move on to the next stage” (P001AN)*, while another remarked, *“I think that certain interfaces could certainly be improved” (P010AN)*. Across both training and design, respondents highlighted the importance of using simple and appropriate language, rather than overly technical terminology: *“It’s true that finding the right language for older adults is complicated*,* I think*,* for young people” (P015AN)*. For many participants, human support remained essential, with 65% emphasizing the need to maintain both digital and non-digital options: *“You still have to have the option of having human support” (P027AN)*. Finally, 46% underscored the importance of continuous adaptation, that is, accepting the need to “move with the times” and regularly update one’s skills as a way of preserving independence in the face of rapid technological change. As one participant stated, *“Older adults need to make the effort to prepare themselves for this” (P001AN)*.

Overall, the solutions proposed by participants converged on a shared message: the need for tailored, continuous, and accessible support to accompany older adults in their use of digital technologies. These proposals highlight that digital inclusion cannot rely solely on technical improvements but also requires sustained human involvement to guide, reassure, and strengthen users’ autonomy.

### Theme 3 : perceptions of the ethical and societal challenges of digitalisation

Beyond barriers and practical solutions, participants also reflected on the broader implications of service digitalisation. Their experiences informed wider concerns related to equity, the existing choice in using technology, the role of human relationships, and data protection. The following section examines these ethical and societal challenges.

#### Digitalisation: choice or obligation?

Beyond the identified barriers and potential solutions described above, participants’ perceptions of whether they still had a real choice in the face of the widespread digitalisation of services emerged as a central theme in their accounts. Three main positions were identified regarding whether the use of digital technologies is experienced as a genuine choice.

For a large majority of participants (83%), digital use was perceived as imposed and inevitable, given the lack of alternatives considered truly accessible. As one respondent stated, *“Well*,* I feel sorry for them [people who don’t want to use technology]*,* absolutely. No*,* but it’s impossible to ignore it.” (P004AN)*; while another concluded, *“There is no escape” (P009AN)*. This perception reflected the feeling that services increasingly channel users toward a single, digitally mediated pathway.

Some participants (25%) believed that choice still exists for the moment, but viewed it as fragile and likely to disappear as non-digital options continue to decline. They described the current situation as a transitional phase, in which traditional alternatives remain available but are gradually vanishing. One participant remarked, *“Sooner or later*,* they [the alternatives] will disappear too” (P013AN)*, while another observed, *“We are heading straight for complete digitalisation” (P026AN)*. At the same time, more than half of respondents (58%) felt that alternatives persist in certain domains and that a degree of choice remains, either through access to human services, such as *“In stations*,* you can still find a ticket office” (P010AN)*, or through personal practices, for example, *“People who don’t want to or can’t declare their income online can do so on paper” (P011AN)*. From this perspective, individuals were still seen as able to decide whether or not to engage with digital technologies.

These contrasting perceptions of choice show that digital use is not just a question of individual skills, but is part of an environment where access conditions, available resources and the organisation of services strongly influence older adults’ ability to adopt these tools.

#### Perceived benefits and inconvenients of digitalisation

Many participants recognized the opportunities offered by digitalisation (71%), emphasizing above all its practical benefits. These included the simplification of everyday procedures, such as administrative tasks and transport services: *“I find it convenient when you top up your Navigo pass and you don’t have to queue anymore…” (P036AN)*, or *“It makes life easier. Even taxes are now all digital*,* the tax form arrives and we can access it on our account. It’s already pre-filled*,* so all we have to do is check that the figures are correct. It’s relatively simple and convenient.” (P026AN)*. Participants also highlighted gains in speed and efficiency, for example, *“I don’t know*,* before I used to look things up in a dictionary*,* but now I just have to press a key and I can find the definition of any word*,* or any concept for that matter” (P019AN)*.

Others valued the easier access to services and information aligned with their interests: *“I do a lot more things*,* at least*,* more interesting things in line with my personality and my wishes.” (P009AN)*. Some participants further pointed to improvements in human relationships (35%), believing that digital tools help maintain contact, especially with distant relatives: *“Technology is also designed to bring people closer together*,* because even though I’m here*,* I can see my granddaughter or my grandsons or my children who are far away” (P020AN)*. These perceived benefits contributed to generally positive attitudes toward digitalisation among a substantial proportion of participants (71%), leading some to summarize its impact in simple terms: *“Well*,* listen*,* I mean*,* there are some good things about it*,* right?” (P006AN)*.

Alongside the perceived benefits, many participants also emphasized negative aspects associated with digitalisation. Several mentioned the risk of malfunction (35%), whether due to technical failures, bugs, network outages, or electrical disruptions that can render services inaccessible: *“I don’t understand how no one is asking the question of whether we are all dependent on the electrical system” (P027AN)*.

Concerns about the deterioration of human relationships were also prominent (58%). While some viewed digital technologies as facilitating social ties, others felt that they contribute to a loss of direct contact, which they described as warmer, more understanding, and more reassuring: *“No*,* what I really miss is human contact. When you have a problem*,* being able to sit down with someone who can explain things to you” (P044AN)*. This ambivalence shows that participants saw digitalisation both as an opportunity and as a loss for social life.

For some participants, this perceived decrease in direct human contact was accompanied by fears of human–machine replacement (29%), understood as the progressive substitution of human roles by automated devices or fully digitalized services, with implications for employment and interpersonal relations. As one respondent illustrated, *“When you used to go shopping*,* you dealt with someone you knew*,* but now you just scan your own items” (P008AN)*.

Taken together, these concerns led many participants to describe digitalisation as detrimental or regrettable (65%), sometimes in reference to past practices: *“No*,* for me*,* it’s a major step backwards” (P017AN)*. Finally, some expressed apprehension about a future loss of control (27%), associated with the rapid pace of technological change and the development of artificial intelligence. The prospect of increasingly autonomous systems that are difficult to understand or control raised concerns about future digital practices: *“I think we’re heading straight for it. Apparently*,* there are already robots that provide personalized responses even though they haven’t been programmed to do so. So you see? That means it’s starting” (P024AN)*.

#### Perceptions of digital security

Consistent with their views on digitalisation more broadly, participants expressed diverse perceptions regarding digital security. Some reported limited concern (44%), adopting a position of general trust and considering that their personal data were unlikely to be of interest. As one participant stated, *“Well*,* personally*,* no [I do not have a concern about security]. I’ve got nothing to hide*,* I don’t do anything stupid*,* nothing at all*,* so no.” (P008AN)*, while another added, *“I’m not too concerned [about digital security]*,* even if that might be a mistake.” (P010AN)*.

However, for the large majority of participants, data confidentiality was described as a sensitive issue (90%) and, in some cases, a source of anxiety. Concerns covered a wide range of perceived risks, including identity theft or misuse of personal data: *“when you hear about all the identity thefts being carried out by malicious individuals. So we might ask ourselves*,* should we put everything online?” (P002AN)*; account hacking and online scams; unintentional exposure on social media: *“Oh yes*,* I don’t want the whole of France to know where I’ve gone on holiday and all that stuff with TikTok and whatever else there is. On Facebook and all that” (P017AN)*; and the possibility of children accessing inappropriate content. Several participants also referred to the impression that online activities are monitored and used for commercial purposes, particularly through targeted advertising. One participant explained, *“If I search for a sofa [to buy]*,* in the next three days I get 36*,*000 ads about sofas. So they know that one day we were interested in a sofa*,* and they try to sell us one. There’s a bit of pressure there.” (P023AN)*.

Overall, concerns related to the security of banking data in the use of digital services were the most frequently mentioned. Even among participants who otherwise reported general confidence, this remained a sensitive area: *“For me*,* the main concern is banking. For now*,* with other services*,* I don’t really see any risk*,* and that seems fine.” (P002AN)*. Another participant similarly noted, *“Well*,* do I really have a lot to hide? As long as they don’t hack into my bank account to take my pension [retirement allowance]*,* I tell myself it’s fine. But I can’t say I’m indifferent to it.” (P015AN)*.

Beyond these security concerns, which ranged from relative confidence to pronounced worries about data protection, several participants also questioned the increasing presence of digitalisation across many areas of everyday life.

#### Justifications for widespread digitalisation

The interviews revealed several perceived justifications for the expansion of digitalisation. Some participants primarily framed it as a process of progress (69%), linked to technological innovation and the idea of societal advancement: *“What motivated it [digitalisation]? Well*,* it’s the normal progress of all centuries. There has been progress in the last century” (P007AN)*. This view was echoed by others who placed digitalisation within a broader historical trajectory: *“Once something new has been invented*,* that’s the meaning of history. It’s like Gutenberg’s printing press*,* then radio*,* then television*,* and after that*,* it’s progress. There’s nothing we can do about it.” (P032AN)*.

Others emphasized arguments related to efficiency, particularly time savings (67%), for example, *“Well*,* speed is useful. It saves you from having to travel for many things” (P005AN)*. Environmental considerations, such as reducing paper use, were also mentioned (17%), although some participants questioned the validity of these claims: *“Apparently*,* we pollute quite a lot by using computers and all that. No*,* I don’t know if it’s really that much better” (P001AN)*.

Some participants also mentioned economic reasons (35%), believing that digitalisation is driven by cost or profitability considerations for organisations: *“So I imagine there’s a financial motive for companies [behind digitalisation]” (P010AN)*, or *“I think [the reason behind digitalisation] it’s the priority given to money*,* or rather cash*,* over… we see it in all areas” (P012AN)*. Overall, even when participants identified what they considered logical explanations for the digitalisation process, several nevertheless questioned their legitimacy, expressing doubts about whether these justifications were fully convincing or fair.

### Statistical analysis

To complement the qualitative findings and explore the potential influence of sociodemographic characteristics, particularly age, on participants’ responses, exploratory quantitative analyses were conducted. These analyses were intended to identify possible tendencies in the data rather than to formally test predefined hypotheses. This section presents the results of (1) chi-square tests comparing subcode mention frequencies between age groups (under 75 vs. 75+), and (2) Spearman’s correlation analyses examining associations between participants’ self-reported ease with technology and sociodemographic variables.

Two subcodes showed differences between age groups that reached statistical significance in this exploratory analysis. A difference on the frequency of the subcode *work* (use of digital tools for professional purposes), was observed between groups, being mentioned more frequently by participants under 75,, χ²(1, *N* = 48) = 5.30, *p* = 0.021, with a moderate effect size (φ = 0.33). The subcode *delegation of the task* (entrusting certain digital tasks to others within one’s social circle), mentioned more frequently by participants aged 75 and over, also showed a difference, χ²(1, *N* = 48) = 5.00, *p* = 0.025, φ = 0.32.

Two additional subcodes were close to the significance threshold but did not reach the conventional α = 0.05 level. This was the case for *technical failures* (difficulties linked to malfunctioning devices or platforms), mentioned more frequently by the group aged under 75, χ²(1, *N* = 48) = 3.80, *p* = 0.051, φ = 0.28, and *affordability* (financial cost as a barrier to digital accessibility), which tended to be cited more often by participants under the age of 75, χ²(1, *N* = 48) = 3.67, *p* = 0.056, φ = 0.28.

Spearman’s correlations did not show a statistically significant association between age and self-reported ease with technology (ρ = −0.20, *p* = 0.18), suggesting that in this sample, age was not clearly related to perceived ease of using technology. In contrast, self-reported ease with technology was positively associated with socio-educational level (ρ = 0.49, *p* < 0.001), suggesting that higher educational attainment was linked to greater ease with digital tools. An association was also observed between self-reported ease with technology and socio-professional category (ρ = −0.36, *p* = 0.011), indicating potential differences across occupational groups.

No statistically significant association was observed between self-reported ease with technology and living environment (ρ = −0.04, *p* = 0.77), when comparing participants living in urban vs. rural areas. However, this result should be interpreted with caution due to the very small number of participants residing in rural areas, as the sample was predominantly composed of individuals living in large urban areas with relatively good access to digital infrastructures and services.

As these analyses were exploratory and based on a relatively small sample, the results should be interpreted with caution. They primarily serve to highlight potential patterns that could be examined more systematically in future studies with larger samples.

## Discussion

This study examined how French older adults approach the ongoing digitalisation of everyday services, identified perceived barriers to digital accessibility, and explored related ethical and societal concerns. It also aimed to provide recommendations for designing and deploying digital services that are more accessible to older adults, thereby limiting situations that generate difficulties, misunderstandings, or exclusion.

### Principal findings

#### Digital approaches and experiences

Consistent with studies showing that most older adults now use at least one digital device [3, 61], all participants in our sample reported using a mobile phone and, for some, additional devices such as computers or tablets. However, as documented in the literature, usage remained concentrated on activities perceived as essential or directly useful, such as communication, information searches, administrative procedures, and entertainment. Our qualitative analysis suggests that usage is maintained when older adults identify clear and meaningful benefits in doing so. Such a practical and selective approach is consistent with prior work noting that, unlike younger age groups who engage digital technologies for diverse leisure, social, and exploratory activities, older adults tend to focus on functionalities that serve immediate and concrete needs [3, 29]. This pattern corresponds with theoretical models that highlight perceived usefulness and perceived ease of use as key determinants of adoption among older adults [33, 47]. These findings are consistent with prior research conducted in various national contexts, which similarly reports that older adults tend to adopt a pragmatic and selective use of digital technologies, primarily oriented toward functional needs [3]. However, some studies conducted in other cultural and infrastructural contexts suggest that patterns of use and perceptions of digitalisation may vary depending on access conditions, welfare systems, and cultural norms related to technology [54, 55].

Participants described multiple difficulties related to the use of digital services that closely reflect well-documented barriers in the literature, including lack of information and support [3, 7], complexity of interfaces [7], technical malfunctions, and limited pedagogical guidance when learning to use technologies. The notion of habit and repetition emerged as particularly important. Many participants expressed apprehension toward unfamiliar tools or procedures, an observation consistent with studies on technostress and aging [36, 62]. Repetition and gradual familiarisation therefore appear to be central mechanisms for building confidence and competence. When tasks or interfaces were not immediately understandable, participants frequently relied on family members, professionals, or trusted intermediaries. However, such support was not always available, and many expressed a desire to remain independent in order to avoid “bothering” others. This reflects WHO [63] recommendations and the work of Vaportzis et al. [26] on the importance of designing technologies that support feelings of self-efficacy. WHO [63] recommendations advocate for devices that enable gradual and understandable use of digital services, for example through step-by-step guided tutorials, adaptable pacing, and error tolerance. In their study, Vaportzis et al. [26] show that older participants particularly appreciate technologies and support mechanisms that encourage repetition, simple explanations and learning without pressure, thereby helping to build confidence and reduce feelings of dependence on those around them. 

As found in other studies [64–66], older adults in our study mobilised diverse strategies to manage encountered difficulties when using digital services, including seeking human assistance, delegating to a relative, turning to in-person service counters, self-learning, or repeated trial-and-error. These strategies illustrate a constant negotiation between the desire for independence and the constraints encountered during technology use, a dynamic documented in past research [26, 67]. Some participants expressed a preference for performing tasks independently while simultaneously feeling dependent on others, reflecting what has been described as technological vulnerability: an unwanted dependence accepted due to limited alternatives, as identified in studies on the integration of existing technologies for aging populations [68, 69]. Taken together, these findings underscore the importance of accessibility and usability when designing digital services for older users.

#### Barriers to accessibility and possible solutions

The results indicate that participants generally perceive digitalisation as unavoidable, although their views on having a genuine choice in using digital services differ. Some considered digital procedures to be imposed, particularly in administrative or banking domains, which is consistent with observations by Friemel [3] and van Deursen and Helsper [70] on the perception of obligation to go digital among older adults. Others acknowledged that alternatives still exist but questioned their sustainability in the context of expanding digitalisation. Finally, several participants identified physical counters or assistance from relatives as viable alternatives, consistent with studies reporting that autonomy can be maintained when non-digital options are preserved [64, 67]. In particular, Barbosa Neves et al. [64] showed that older adults are more likely to engage with digital technologies when these are experienced as complementary to, rather than replacements for, existing forms of support, allowing them to preserve a sense of autonomy and social connection. Similarly, Seifert et al. [67] highlighted that the disappearance of non-digital alternatives can exacerbate both digital and social exclusion, whereas maintaining offline options helps older adults cope with digital demands without feeling forced or marginalised. In summary, older adults’ engagement with digital services seems to depend in part on whether they feel they have a choice, and this perceived choice is influenced by the presence or absence of non-digital options. While digital technologies are often framed in the general population as tools of convenience, optimisation, and expanded possibilities, our findings suggest that older adults more frequently experience digital services as imposed infrastructures that condition access to essential services rather than simply offering additional options, reflecting persistent generational differences in usage patterns and digital autonomy [29, 70].

Perceived barriers to accessibility further suggest that digitalisation may reinforce existing inequalities, particularly for individuals who are less experienced with technology, have lower levels of education, or live in rural areas. As reported in prior studies, barriers include interface complexity, the rapid pace of technological change, lack of appropriate informational support, and material or financial constraints [3, 27, 71]. These barriers contributed to reported feelings of exclusion, sometimes leading older adults to reduce or discontinue their use of digital services, a phenomenon documented in previous research [68].

In response to these barriers, participants proposed several solutions that align with existing literature on digital support for older adults. Tailored training emerged as a preferred strategy for improving digital confidence and competence [26, 36, 65], while individualised human assistance was viewed as essential for resolving specific difficulties and adapting guidance to personal needs. The work of Vaportzis et al. [26] shows, for example, that the most effective training courses are those based on progressive learning, focused on concrete, everyday uses, and which give older people time to experiment without fear of making mistakes. Kim et al. [36] also show that the gradual learning process involved in using a tablet relies as much on repetition and hands-on experience as it does on adapting familiar methods, highlighting the importance of a guided learning phase and taking into account users’ existing strategies to promote technology adoption among older adults. Similarly, Quan-Haase et al. [65] emphasise that the adoption of technologies is part of existing routines and relies heavily on personalised explanations tailored to users’ individual goals. In addition, participants emphasised the importance of inclusive interface design, including clear instructions, simplified vocabulary, and adapted ergonomics, to reduce errors and frustration [67]. Similarly, Seifert et al. [67] show that interfaces that are difficult to read, overly complex or insufficiently adapted to the abilities of older users contribute to both digital exclusion and feelings of social exclusion, emphasising that poor design choices can be as significant a barrier as a lack of technical skills. These suggested solutions are consistent with previous recommendations in the literature indicating that a combination of accessible digital tools and human assistance can reduce inequalities in access and support autonomy among older adults [68, 69]. In particular, the systematic review by Peek et al. [68] highlights that acceptance of technologies for aging in place depends not only on perceived usefulness and usability, but also on the availability of adequate support and the reassurance that help remains accessible when difficulties arise. Similarly, Marston and van Hoof [69] emphasise that digital solutions must be embedded within supportive social and physical environments, arguing that technology alone cannot foster autonomy without accompanying human and organisational resources.

#### Perceptions of the ethical and societal challenges of digitalisation

The feelings expressed by participants confirm previous observations regarding the emotional dimension of digital use among older adults [72]. In particular, LeRouge et al. [72] showed that emotional responses play a key role in technology adoption among older adults, with feelings of confidence, perceived control and readiness facilitating engagement, while anxiety, frustration and low self-efficacy act as significant barriers, especially when technologies are perceived as complex or insufficiently supported. Our results extend this perspective by showing that experiences range from feelings of competence and lack of concern to reluctance, frustration, fear, abandonment, and determination. This variability has been documented in theories of social ageing [73], where success with digital tools can reinforce competence, while difficulties can be perceived as exclusion or as a reminder of aging.

Participants in our study recognised several benefits associated with digitalisation, including convenience, speed, simplification of tasks and access to new possibilities, confirming evidence on the perceived usefulness of technologies among older adults [7, 33]. They generally did not dispute the idea of technological progress, a finding also reported in prior empirical work examining older adults’ attitudes toward digital technologies in different national contexts [28]. At the same time, they expressed concerns about the reduction of human interaction, the perception that certain human roles are being replaced by machines, and the possibility of losing control over technology, particularly with the expansion of artificial intelligence. These concerns are consistent with research addressing tensions between technological progress and perceived dehumanization [74–76]. Several authors have shown that although technological tools can facilitate certain exchanges, they may also alter the perceived quality of interpersonal relationships, particularly among older adults in situations of vulnerability or social fragility [77, 78]. Fear related to AI also emerged in our study, likely reflecting the current socio-technical context characterised by the rapid spread of generative AI tools.

This combination of perceived benefits and concerns suggests that many older adults assess digitalisation not only in terms of efficiency, but also through its implications for social relationships and human presence. In this regard, improving the functional and financial accessibility of digital devices appears to be an important factor for sustaining engagement among older adults.

Security and privacy were identified as major concerns, consistent with previous studies [3, 79]. Indeed, Friemel [3] shows that concerns about security and privacy are strongly associated with lower levels of digital engagement among older adults, particularly when combined with limited digital skills and confidence. Similarly, Tsai et al. [79] highlight that fears related to data security and making irreversible errors can inhibit exploration and learning, underscoring the role of trust and perceived safety in older adults’ digital practices. However, our results indicate that concerns were most pronounced regarding banking information, whereas other personal data were perceived as less sensitive. Participants also mentioned risks such as surveillance, hacking, online scams, targeted advertising, and unwanted exposure of personal information, highlighting a discrepancy between objective risks and perceived vulnerabilities. These findings reinforce that data protection is a central dimension of technology adoption and should be incorporated explicitly into digital services aimed at older adults [42].

Participants also identified familiar justifications for digitalisation, including progress, environmental benefits, efficiency, and economic considerations. These explanations align with those frequently advanced in public policy [80]. Indeed, the OECD emphasises that digitalisation is commonly justified in public discourse by arguments related to efficiency gains, cost reduction, service optimisation, and environmental sustainability, particularly in the context of ageing societies and constrained public resources [80]. However, some participants questioned the validity of these arguments, reflecting critiques found in analyses of techno-solutionism [81]. In this perspective, Greenfield [81] argues that techno-solutionist narratives tend to present digital technologies as neutral and inevitable answers to complex social issues, thereby downplaying their social, ethical, and political implications. These discussions indicate that older adults are not passive recipients of digitalisation, but develop their own interpretations regarding its motivations and limitations. From an ethical and societal standpoint, the findings suggest that digitalisation is experienced not only as a technical shift but also as a process that affects autonomy, responsibility, and the role of human relationships in everyday services.

### Age and digital inequalities: limited differences in our sample

Many studies report marked differences in digital usage, attitudes, and skills according to age, particularly between adults above and below 75 years [3, 7, 70, 82]. In contrast, our analysis revealed few age-related variations within the present sample. An exploratory comparison between participants under 75 and those aged 75 and over showed that only two subcodes differed significantly between age groups. The limited differences observed appeared to be linked more to situational factors rather than generational ones. For example, the subcode *work*, referring to the use of digital tools for professional activities, was more frequently cited by participants aged 60 to 75, likely reflecting ongoing professional activity. In this case, the observed variation may reflect occupational context and life circumstances rather than differences in attitudes toward digital technologies based solely on chronological age.

In addition, no statistically significant correlation was found between age and self-reported ease with technology. This result may partly be explained by the characteristics of the participants, who were predominantly highly educated, from skilled professional backgrounds, and living in urban areas, factors known to mitigate the effect of age on digital usage [27, 70]. In our data, individuals reporting greater ease with technology also tended to have higher socio-educational levels and higher socio-professional categories. Taken together, these observations suggest that, in certain contexts, digital inequalities among older adults may be influenced not only by chronological age but also by social, educational, and contextual factors.

### Recommendations for more accessible digital services

Based on the analysis of the data gathered during the interviews, we developed a set of practical recommendations to support the design and implementation of digital services that are accessible to older adults. These recommendations are intended for public and private organisations that design or deploy digital services, as well as for front-line professionals such as digital mediators, social workers, administrative staff, and reception personnel. The objective is to promote forms of digitalisation that respect the abilities, preferences, and learning pace of older adults.

1. Simplify interfaces and standardise navigation

The results highlight the importance of simpler, more readable, and more consistent interface design. Navigation paths should involve limited steps and reduced visual complexity, and technical terminology should be avoided in favour of accessible language in order to decrease cognitive load and support users’ sense of control. Improved graphic and functional consistency across platforms (e.g., applications, websites, service terminals) could also reduce frequently reported difficulties. Involving multiple stakeholders, including end-users, through participatory design approaches may contribute to services that are more responsive to diverse accessibility needs.

2. Maintain non-digital alternatives to preserve choice

Participants emphasised the need to maintain genuine non-digital alternatives. Maintaining physical service counters, accessible telephone lines, and the option to complete procedures by post or by appointment is essential to ensure that digitalisation is not perceived as imposed or exclusionary. This diversity of access modalities contributes to preserving the sense of choice, which was highly valued in participants’ accounts.

3. Strengthen human support and digital mediation

Human support represents a key mechanism in facilitating digital engagement. Trust was primarily associated with interactions involving available, competent, and attentive intermediaries. Strengthening digital mediation initiatives, training reception staff, and involving local structures (e.g., libraries, town halls) could facilitate gradual adoption of digital tools and reduce anxiety linked to technology.

4. Provide tailored and progressive training opportunities

Older adults expressed a need for training that is adapted to their pace and experience. Such training should be accessible, free of charge, progressive, delivered through multiple channels, and avoid technical vocabulary. Regular group workshops, complemented by individual sessions for complex situations, may support learning and increase perceived competence. Training approaches that reduce apprehension and reinforce self-efficacy appear particularly relevant.

5. Preserve human interaction in service delivery

The findings indicate that preserving opportunities for human interaction remains important. When digital systems fully replace direct human contact, some participants perceived this change as a reduction in service quality or as an indication that human roles were being replaced by automated systems. Including options for direct contact and providing clear guidance on the use of automated tools may mitigate these concerns.

6. Communicate clearly about security and privacy

Concerns regarding data security and confidentiality require attention. Institutions should communicate in a transparent and accessible manner about the protective measures implemented and clarify the nature of risks, particularly those related to fraud and phishing. Facilitating the configuration of privacy settings and limiting unnecessary collection of personal data could further support user trust (Table 4).

**Table 4:** Recommendations for more accessible digital services for older adults

Recommendation	Problem addressed	Proposed solution	Example
Simplify interfaces and standardise navigation	Many older adults experience high cognitive load and confusion due to complex layouts, inconsistent steps, and technical jargon	Use plain language, reduce steps, standardise icons and menus, and remove unnecessary technical terminology	A national health insurance portal redesigns its interface so that older users can complete key procedures (e.g., reimbursement claims) through fewer, clearer steps
Maintain non-digital alternatives to preserve choice	Older adults without devices, skills, or internet access may feel forced to use digital tools, leading to exclusion	Maintain in-person counters, telephone lines, and paper-based options	A tax administration allows older adults to file returns by phone or in-person appointment, in addition to online filing
Strengthen human support and digital mediation	Older adults often need trusted support to understand and carry out online procedures	Expand digital mediation networks, train frontline staff, and involve local community organisations	Community centres and libraries offer assisted sessions helping older adults complete online healthcare, pension, or transportation services
Provide tailored and progressive training opportunities	Many training programmes are too fast, too technical, or not adapted to older adults’ learning pace	Offer free, paced training with age-adapted vocabulary and step-by-step learning	Adult education programmes provide weekly beginner workshops for older adults on email use, appointment booking, or secure messaging
Preserve human interaction in service delivery	Full automation can reduce trust and make older adults feel abandoned or unable to ask questions	Provide hybrid systems combining digital tools and human contact points	Hospitals or public offices offer both touchscreen check-in kiosks and staffed reception desks for older adults who need assistance
Communicate clearly about security and privacy	Concerns about scams, fraud, and data misuse are particularly strong among older adults, reducing trust	Explain risks and protections in simple terms, reduce unnecessary data requests, and offer help configuring privacy settings	Banks and public institutions distribute easy-to-read guides on phishing, secure payments, and two-factor authentication targeted at older adults

### Limitations of the study

The main limitation of this study concerns the representativeness of the sample. First, part of the recruitment was conducted via email, which presupposes access to digital equipment and a minimum level of digital competence. This approach may have excluded older adults who are less equipped, less digitally confident, or opposed to digital technologies, thereby under-representing profiles that could have further nuanced the findings.

Second, despite efforts to include participants from diverse geographic regions and living environments, the majority of respondents lived in urban areas, particularly in the Paris region. Older adults residing in rural environments often experience more limited digital infrastructure, reduced access to support, and different perceptions of digital technologies [83]. In the present study, most participants lived in areas where access to digital infrastructures, public services and technological support may be more readily available. This urban context may have influenced participants’ experiences of digitalisation and may limit the transferability of the findings to rural settings, where infrastructural constraints and access to support may differ.

Third, although the sample included 48 participants, it contained a high proportion of individuals from intermediate or higher socio-professional categories with relatively high levels of education. Given that technology appropriation varies according to educational and cultural capital [70], this composition may under-represent segments of the older population with lower socio-economic status or lower educational attainment. In addition, other socio-demographic factors that may influence access to and use of digital technologies, such as migration background or levels of digital health literacy, were not specifically collected in this study and could not be examined.

A further limitation relates to the geographical context of the study, which was conducted in France and, more broadly, situated within a European policy and infrastructural landscape. Digital infrastructure, support mechanisms, and institutional practices vary considerably across regions globally, meaning that comparisons with other contexts (e.g. North America, Asia, or low- and middle-income countries) may be limited.

Beyond issues of representativeness and geographical context, the study also presents methodological limitations. Data were self-reported, which may introduce recall bias (i.e., inaccuracies in participants’ recollection of past experiences or behaviours), particularly when referring to past digital experiences or learning trajectories. In addition, self-reported digital skills and attitudes may be influenced by self-report bias (i.e., tendencies to over- or under-estimate one’s own abilities or behaviours when answering questions), which may result in discrepancies between participants’ perceived and actual levels of competence or comfort with digital tools. Moreover, neither interview transcripts nor findings were returned to participants for validation (member checking). Although ambiguities were clarified during the interviews through follow-up questions, participants did not have the opportunity to review the researchers’ interpretations afterwards, which may limit the extent to which the findings fully reflect their perspectives.

Finally, the study employed a cross-sectional design, which captures perceptions and practices at a single point in time. This approach limits the ability to analyse changes in digital use, attitudes, or barriers as technologies evolve or as individuals gain experience. Longitudinal studies would be needed to better understand how older adults’ digital practices and perceptions develop over time.

### Strengths of the study

Despite these limitations, our study has several strengths that should be noted. First, it examines a contemporary topic in a context where practices, tools, and policies are evolving rapidly, providing empirical data on older adults’ experiences with digitised services at a relevant moment. Second, the use of qualitative interviews allowed for the collection of detailed accounts regarding practices, perceptions, and difficulties that are not easily captured through quantitative instruments.

Third, the sample included older adults aged 60 to 96 years, with varied sociodemographic and digital profiles, supporting a more differentiated description of digital practices within this population. Fourth, the analytical process involved independent coding and consensus discussions, contributing to the rigour and consistency of the qualitative analysis. Fifth, the triangulation of qualitative findings with exploratory quantitative analyses offered complementary insights regarding potential associations between sociodemographic variables and digital familiarity.

Finally, the study adopted a user-centred perspective by foregrounding the lived experiences of older adults. This approach supports ecological validity and provides transferable insights for actors involved in designing or mediating digital services intended for older users.

## Conclusion and future work

This study examined the experiences, expectations, and concerns of older adults living in France regarding the digitalisation of everyday services. The findings reveal a tension between the perceived benefits of digital tools (e.g., speed, convenience, ease of access to information) and the obstacles that hinder their use, whether technical, affective, or linked to a reduction in human interaction. These results underline the need to design services that are accessible, intuitive, and supported, in order to promote voluntary rather than imposed use.

Although digital services now play a central role in domains such as administration, banking, transport, healthcare, and communication, there are still limited practical recommendations on how to adapt them to older adults. This study provides an overview of their perspectives and highlights several avenues for improving accessibility and acceptability. Further work is needed to translate these insights into standardised accessibility frameworks and operational deployment strategies.

In light of the limitations identified, future research should include older adults with more diverse sociodemographic profiles, particularly those living in rural areas or with lower levels of education, in order to better capture structural and contextual barriers. Comparative studies across European contexts would also help clarify how policy environments and infrastructures shape experiences of digitalisation. In addition, longitudinal or repeated-measures designs would make it possible to assess how practices and attitudes evolve over time, while mixed-method approaches combining interviews with behavioural observations or usage data could reduce reliance on self-report and recall.

Finally, intervention studies evaluating training programmes, mediation mechanisms, or interface redesigns would provide evidence on effective levers for improving digital accessibility and maintaining autonomy. By addressing these gaps, future research can support the development of inclusive and evidence-based frameworks that ensure technological developments benefit older adults rather than exacerbate existing inequalities.

## Supplementary Information

Supplementary Material 1.

## Data Availability

The datasets generated or analyzed during this study are available from the corresponding author on reasonable request.
