# Supplementary material for: Perspectives of French community-dwelling older adults on the use of digital services: a qualitative study based on interviews
Source: BMC Geriatr. 2026 May 9;26:757. doi: 10.1186/s12877-026-07511-w (PMC13217671; doi:10.1186/s12877-026-07511-w)
Supplement: Supplementary file 1 — Supplementary Material 1. [file 12877_2026_7511_MOESM1_ESM.docx]

**Annex 1 : COREQ grid**


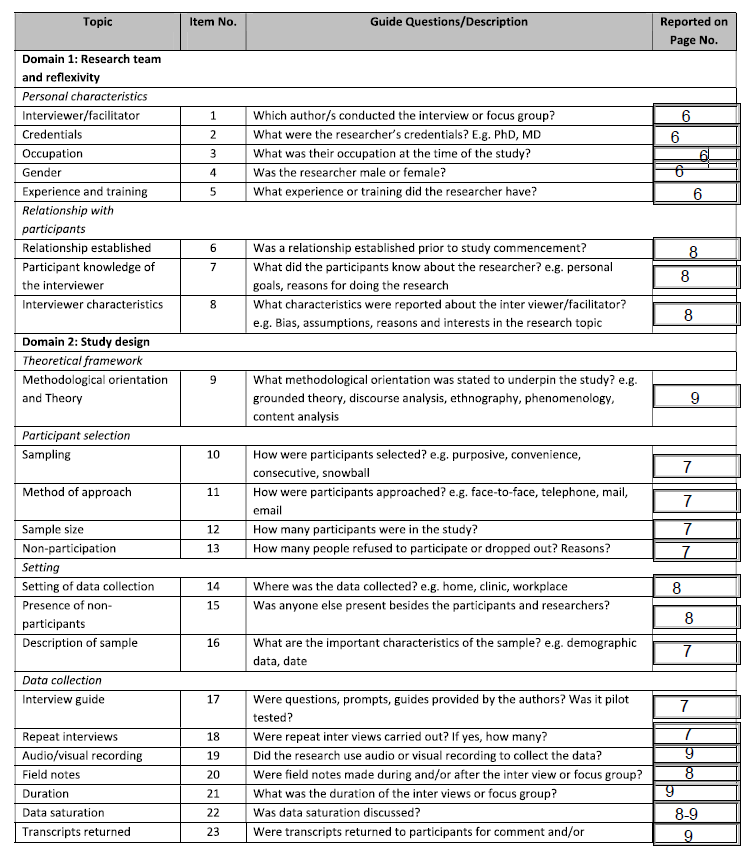


**
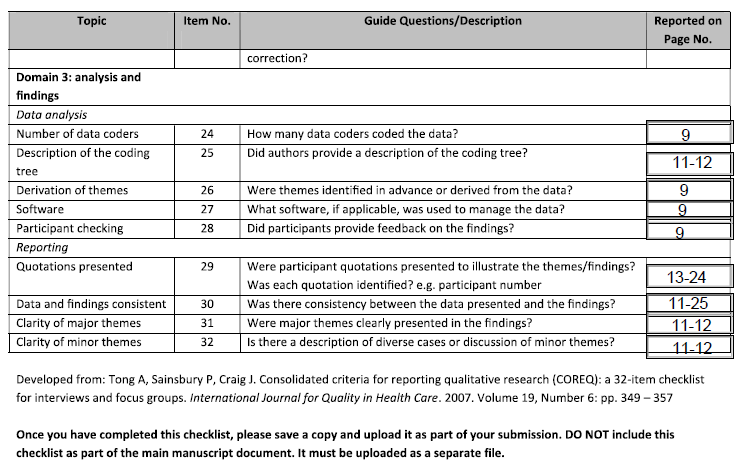
**

**Annex 2 - Interview guide**

1. What information technologies do you use in your daily life? (e.g. tablet, computer, ATM, smartphone, shopping terminals, etc.)

2. What services do you access using these devices? (e.g. communication, games, email, Doctolib, health insurance, taxes, shopping, banking transactions, etc.)

3. How often? (Frequently, occasionally, when I have to, never, etc.) In what type of environment do you use these technologies? (e.g. home, station/airport, healthcare facility, shopping, catering service/QR code, etc.)

4. Do you find these digital services easy to use? (Interface, instructions, purpose, etc.) Why?

5. If so, what difficulties do you encounter when using digital services? (Is the information on the screen difficult to understand, are there too many steps, is there not enough guidance?)

6. How do you feel when you are unable to use these technologies?

7. Have you ever needed outside help to use these technologies? Were you able to find this help? If so, how did you find it? (Did you need someone else's help?)

8. Do you know of any solutions (other than humans) that can help you overcome difficulties related to using technological tools? Have you ever used them (e.g., virtual agents, voice-activated assistants, chatbots, guides)? If so, how was your experience?

9. How do you feel when the digital service does not work as it should (e.g. bugs, resets, screen freezes, error messages)? How do you deal with the problem? (e.g. abandonment, training)

10. What is your opinion/view on the increasing digitalisation of services (e.g. banking transactions, taxes, social security, etc.)? a. If positive: Why? b. If not: Why? What alternative solution would you suggest? (Technological mediation)

11. In your opinion, what has motivated the digitalisation of many services?

12. Do you feel that you have a choice/that there is always a non-digital solution? Do you think there is another alternative for people who do not wish to use them?

13. In your opinion, is this digitalisation beneficial, desirable, risky, regrettable, etc.? Could this trend lead to the exclusion of certain groups?

14. Do you feel that digital services are designed with older people in mind? Why or why not?

15. Do you feel you have everything you need to use digital services properly?

16. Do you feel that digital services have improved your quality of life? If so, in what way? If not, why not?

17. What suggestions would you make to improve the accessibility of digital services for older people?

18. Are you concerned about the confidentiality of your personal information when using digital services?

**Annex 3 - Definition of each subcode with an associated verbatim**

| **THEME** | **CODE** | **SUBCODE** | **Définition** | **Verbatim exemple** |
| --- | --- | --- | --- | --- |
| **(1) How older adults describe their everyday experiences with digital services, including their patterns of use, adaptive strategies, and attitudes toward these technologies** | **Reasons for use** | **Work** | Use of digital technology for professional activities or activities related to a previous job (e.g. writing, document management). | ‘So it helps me work, it helps me access the things I work with, whether it's email, writing, word processing, or document software.’ (P030AN) |
|  |  | **By obligation** | Use of digital technology motivated by constraints, lack of alternatives or administrative requirements. | ‘When there is no other option, of course I use technology.’ (P011AN) |
|  |  | **Communication** | Use of digital technology to maintain social connections (emails, messaging, calls, video conferences). | ‘I only really use it for a few emails, but very, very few, lots of messages, yes to messaging and then phone calls.’ (P020AN) |
|  |  | **Informational** | Use of digital technology to search for information or various content via the Internet (news, health, travel, culture, etc.). | ‘For me, it's for all my research, whether it's about travel, geography, medicine, or anything else. I look up everything and anything.’ (P003AN) |
|  |  | **Administratives formalities** | Use of digital technology to carry out administrative or practical tasks online (taxes, post office, shopping, bookings, etc.). | ‘I do my taxes online, and I used to do all my post office business online.’ (P025AN) |
|  |  | **Entertainment** | Use of digital technology for leisure or relaxation activities (games, reading, videos, online newspapers). | ‘Ah, well, my computer, for pleasure, because I read the newspaper on it and play games.’ (P021AN) |
|  | **Facilitating factors** | **Ease of use** | A feeling of simplicity or comfort when using digital tools. | ‘even in general, I can do it, no problem.’ (P033AN) |
|  |  | **Being used to** | Familiarity with digital tools acquired through repeated use. | ‘It's easy. It's easy, out of habit, the first few times, certainly less so.’ (P008AN) |
|  |  | **Good ergonomics** | Positive perception of the interface or design of a tool, facilitating navigation and use. | ‘Yeah, yeah, I still use the tablet, it's quite user-friendly, it's flexible.’ (P034AN) |
|  | **Limiting factors** | **Difficulty of use** | Difficulty related to age, a feeling of inability, or lack of skills to use digital tools. | ‘Given my age and my ability, I think I really need to start from scratch, and it might not be easy for me to take it all in.’ (P001AN) |
|  |  | **No being used to** | Difficulty related to new technologies or unfamiliar features. | ‘Well, as soon as I step outside my comfort zone, I feel a bit lost.’ (P019AN) |
|  |  | **Technical failures** | Difficulties related to devices (malfunctions, frequent replacement, incompatibilities). | ‘They keep changing our equipment, and we keep ending up with the same problems. And we just get on with it. I don't know how much time it takes here, but I can say that I spend 10% of my week dealing with IT issues. That's not normal.’ (P030AN) |
|  |  | **Lack of user support and documentation** | Difficulty related to insufficient explanations or guides for understanding how digital tools work. | ‘So there is a lack of written information on how to use these types of devices.’ (P002AN) |
|  |  | **Poor ergonomics** | Difficulty related to the complexity of interfaces, the multiplicity of steps, or confusion in navigation. | ‘There are many steps, yes. And as a result, I forget to confirm.’ (P001AN) |
|  | **Coping strategies** | **Human assistance** | Seeking help from someone (family member, professional, friend) to learn how to use digital technology. | ‘Absolutely, we have to call on personal and human assistance, human assistance.’ (P002AN) |
|  |  | **Delegation of the task** | The act of entrusting certain digital tasks to others, particularly through organisation within one's social circle. | ‘I use the tablet more than the computer because it's more my husband's.’ (P004AN) |
|  |  | **Dependency** | Feeling of being unable to use digital technology without external assistance | ‘Fortunately, the IT department came to help. Otherwise, I wouldn't have been able to manage.’ (P032AN) |
|  |  | **Preference for human service** | Choice to prioritise human contact or face-to-face interactions over digital tools | ‘I prefer to ask someone for information.’ (P015AN) |
|  |  | **Seek for non digital alternative** | Seeking or maintaining non-digital solutions (counter, telephone, paper mail). | ‘To buy a train ticket, I prefer to go to the station and actually speak to a ticket clerk.’ (P011AN) |
|  |  | **Learning/Adaptation** | Process of learning, practising or gradually adjusting to the use of digital tools. | ‘Well, after a while, I am able to remember how I did it.’ (P006AN) |
|  | **Attitudes and experiences of digital technology** | **Feeling of competence** | Feeling of personal insecurity when using digital technology, affecting confidence and ease. | ‘I panic as soon as I don't know, as soon as I tell myself I won't be able to do it because I have so many doubts. It's terrible. I get stressed.’ (P003AN) |
|  |  | **Generational gap** | Attributing difficulties encountered to generational factors or ageing. | ‘But yeah, maybe if I were 20 years younger, I would have got used to it and found it easy.’ (P020AN) |
|  |  | **Lack of concern** | Lack of interest or particular concern regarding difficulties in using digital technology | ‘And anyway, that's not my main concern, eh, I sleep well at night.’ (P007AN) |
|  |  | **Feeling at ease** | Feeling of familiarity and comfort in using digital tools. | ‘But I always manage somehow.’ (P025AN) |
|  |  | **Reluctance toward technology** | Reserved or distrustful attitude towards the use of digital technology | ‘I don't dare go [on internet] because I won't understand the words.’ (P039AN) |
|  |  | **Abandonment/Waiver** | Decision to stop using digital technology after experiencing difficulties or a loss of motivation. | ‘Oh well, I give up after a while.’ (P005AN) |
|  |  | **Irritation/Frustration** | Negative emotional reaction to difficulties in use or technological malfunctions. | ‘When I can't get what I want, but it annoys me, what do you want me to say?’ (P031AN) |
|  |  | **Determination to use technology** | Persistence and willingness to learn despite obstacles encountered. | ‘even though I was really on my own in doing it, I ended up succeeding by persevering.’ (P044AN) |
| **(2) Older adults' perceptions of barriers to digital accessibility and possible solutions** | **Barriers to accessibility** | **Digital literacy** | Difficulty of access due to low competence or lack of familiarity with digital tools and environments. | ‘Well, I think there are many problems for people who are not computer literate.’ (P022AN) |
|  |  | **Lack of age-inclusive design** | Difficulty of access due to insufficient consideration of the needs and capabilities of older people in the design and dissemination of digital services. | ‘Oh, so it's difficult for older people to keep up.’ (P007AN) |
|  |  | **Socio-educational level** | Difficulty of access due to inequalities in education, digital literacy or academic background. | ‘But then again, I don't know about people who haven't had a very long education.’ (P006AN) |
|  |  | **Geographical** | Difficulty of access due to uneven Internet coverage and the territorial divide. | ‘Well, I think there really are areas that don't have any internet access at all.’ (P010AN) |
|  |  | **Affordability** | Difficulty of access due to the cost of the hardware, subscriptions or equipment required to use it. | ‘Currently, economic conditions are becoming increasingly difficult, aren't they? And when we tell people, “Well, you have to do this using a computer or something else,” don't we? The famous teleworking from home, well, that already means you have to have a computer. However, at present, computers and all the costs that come with them are not within everyone's reach, whatever we may think.’ (P013AN) |
|  |  | **Feeling of exclusion** | Feeling of exclusion or marginalisation in the face of the increasing digitisation of services and the demands it imposes. | ‘You quickly get left behind, you quickly become isolated.’ (P009AN) |
|  | **Suggestions to improve accessibility** | **Human support** | Human support as a proposed solution to aid learning, understanding and building trust in digital technology. | ‘The alternative is to have a human being, a human being and more. How can I put it? Someone who understands your situation. They must not only be human.’ (P031AN) |
|  |  | **Training** | Training as a proposed solution to strengthen digital skills and promote user autonomy. | ‘It's with a capital T, it's training, you have to train people, not just at home. Yes, because some people can travel, you have to think about that.’ (P002AN) |
|  |  | **Inclusive design** | Inclusive design as a proposed solution to make interfaces more intuitive and accessible to all. | ‘People need to develop in such a way that it is understandable to as many people as possible.’ (P003AN) |
|  |  | **Having the motivation to adapt** | Personal adaptation as a proposed solution for keeping pace with technological and social change. | ‘So yes, but you have to question yourself often because as technology evolves, if you want to continue to be able to manage something, you have to keep up with technology because.’ (P043AN) |
| **(3) Older adults' views on ethical and societal challenges related to digital services, accessibility, digital inclusion and social inclusion** | **Positives aspects** | **Opportunities /Simplification** | Positive perception of digitisation as a source of convenience, increased opportunities and simplification of daily activities. | ‘I do a lot more things, at least more interesting things in line with my personality and my wishes. No, it's an added comfort.’ (P009AN) |
|  |  | **Improvement of sociabilisation** | Positive perception of digitalisation as facilitating exchanges and maintaining social ties. | ‘But when it comes to human relationships, I find that it facilitates relationships and makes it easier to connect with people, for example.’ (P004AN) |
|  |  | **Enjoyment of technology use** | Overall favourable perception of digital technology, associated with a development considered useful or beneficial. | ‘I think that's very good.’ (P022AN) |
|  | **Negative aspects** | **Future loss of control** | Negative perception linked to the fear of technological developments that surpass individuals and are beyond their control. | ‘Apart from new technologies such as ChatGPT, which are a bit frightening. Because they are beyond our comprehension.’ (P005AN) |
|  |  | **Risk of malfunction** | Negative perception linked to the technical vulnerability of digital systems and dependence on their proper functioning. | ‘But the disadvantage is simply that there is a risk of IT system failure, which can occur either due to a lack of power supply or other issues that happen very often.’ (P002AN) |
|  |  | **Loss of human connection** | Negative perception linked to the dehumanisation of exchanges and the disappearance of direct contact. | ‘But what really saddens me is the disappearance of human contact, human relationships.’ (P011AN) |
|  |  | **Digitisation perceived as detrimental** | Nostalgic perception of a past that is considered simpler, more human or more satisfying before the widespread use of digital technology. | ‘I was happier in the eighties. Well, I was younger then, and there wasn't all this technology around.’ (P032AN) |
|  |  | **Risk of human replacement** | Negative perception linked to the replacement of human labour or interactions by technological devices. | ‘Well, first of all, because I think it creates a lot of unemployment. I see it in the shops.’ (P003AN) |
|  | **Perceived choice in digital use** | **Existing choice** | Perception that alternatives exist despite constraints | ‘There is always a solution.’ (P009AN) |
|  |  | **Ephemeral choice** | Perception of a gradual disappearance of traditional non-digital solutions. | ‘It's becoming less and less common. We're heading straight towards complete digitisation.’ (P026AN) |
|  |  | **Imposed use** | Perception of a constraint or obligation to use digital technology without any real choice or alternative. | ‘Oh no, you don't have a choice, you're faced with a fait accompli here. Nowadays, even the smallest things are done online, so no, you don't have a choice.’ (P008AN) |
|  | **Justifications for widespread digitisation** | **Perceived as part of progress** | Justification of digitisation as a symbol of technological progress and modernisation of society. | ‘That's progress. You can't stop progress; that's how it is, from the moment it was conceived. It will be implemented, that's how it is.’ (P024AN) |
|  |  | **More ecological** | Justification for digitisation as a means of reducing paper usage and environmental impact. | ‘There are lots of things that are more on paper now, so in terms of the environment, that's good.’ (P044AN) |
|  |  | **Economic gain** | Justification for digitisation based on the savings it enables, particularly for businesses and institutions. | ‘Ah, I think first of all, there is the lure of financial gain.’ (P017AN) |
|  |  | **Time saving** | Justification for digitisation based on the speed and efficiency it provides in completing tasks. | ‘It's quicker, I think. It saves time.’ (P001AN) |
|  | **Perceptions of digital security** | **Confidentiality risks** | Concerns about personal data protection and fears of a lack of online privacy. | ‘And because we don't really know where our data ends up.’ (P023AN) |
|  |  | **Low concern about digital security** | Confident attitude towards digital security, expressing acceptance of risk or trust in security measures. | ‘No, it's true, I do all my accounts online like that, no, it doesn't worry me. Maybe we'll take the risk.’ (P018AN) |

**Annex 4 - Details of the socio-demographic characteristics of the participants**

| Participant ID | Sex | Age | Living environment | Level of comfort with technology | Years of schooling | Socio-professional category |
| --- | --- | --- | --- | --- | --- | --- |
| P001AN | F | 80 | Urban | Not at all | 12 | 4 |
| P002AN | M | 83 | Urban | Comfortable | 14 | 3 |
| P003AN | F | 75 | Urban | Moderately | 14 | 3 |
| P004AN | F | 83 | Rural | Moderately | 15 | 4 |
| P005AN | M | 85 | Rural | Moderately | 15 | 4 |
| P006AN | F | 77 | Rural | Moderately | 12 | 4 |
| P007AN | M | 84 | Urban | Moderately | 11 | 6 |
| P008AN | F | 85 | Urban | Moderately | 14 | 4 |
| P009AN | F | 84 | Urban | Comfortable | 16 | 4 |
| P010AN | F | 64 | Urban | Comfortable | 17 | 3 |
| P011AN | F | 77 | Urban | Moderately | 14 | 4 |
| P012AN | F | 89 | Urban | Not at all | 15 | 4 |
| P013AN | M | 83 | Urban | Comfortable | 17 | 3 |
| P014AN | M | 90 | Urban | Comfortable | 20 | 4 |
| P015AN | F | 86 | Urban | Moderately | 14 | 4 |
| P016AN | M | 78 | Urban | Comfortable | 11 | 3 |
| P017AN | M | 69 | Rural | Moderately | 17 | 1 |
| P018AN | F | 69 | Rural | Moderately | 11 | 1 |
| P019AN | M | 82 | Urban | Moderately | 15 | 3 |
| P020AN | F | 78 | Urban | Moderately | 12 | 3 |
| P021AN | F | 80 | Rural | Moderately | 12 | 4 |
| P022AN | H | 70 | Urban | Comfortable | 17 | 3 |
| P023AN | F | 73 | Urban | Moderately | 17 | 4 |
| P024AN | F | 68 | Urban | Comfortable | 17 | 5 |
| P025AN | M | 66 | Urban | Moderately | 12 | 3 |
| P026AN | M | 77 | Urban | Comfortable | 17 | 3 |
| P027AN | F | 71 | Urban | Moderately | 20 | 2 |
| P028AN | F | 71 | Urban | Not at all | 5 | 4 |
| P029AN | M | 78 | Urban | Comfortable | 19 | 3 |
| P030AN | F | 64 | Urban | Moderately | 16 | 3 |
| P031AN | F | 70 | Urban | Comfortable | 16 | 4 |
| P032AN | M | 65 | Urban | Not at all | 14 | 5 |
| P033AN | M | 81 | Urban | Moderately | 11 | 2 |
| P034AN | M | 69 | Urban | Moderately | 12 | 4 |
| P035AN | F | 70 | Urban | Comfortable | 16 | 2 |
| P036AN | F | 67 | Urban | Comfortable | 17 | 3 |
| P037AN | F | 72 | Urban | Comfortable | 17 | 3 |
| P038AN | M | 60 | Urban | Comfortable | 17 | 3 |
| P039AN | F | 79 | Urban | Not at all | 9 | 5 |
| P040AN | M | 81 | Urban | Not at all | 11 | 3 |
| P041AN | F | 87 | Urban | Not at all | 15 | 4 |
| P042AN | F | 60 | Urban | Not at all | 17 | 4 |
| P043AN | M | 61 | Urban | Comfortable | 11 | 4 |
| P044AN | F | 66 | Urban | Not at all | 17 | 4 |
| P045AN | F | 86 | Urban | Not at all | 12 | 4 |
| P046AN | M | 96 | Urban | Not at all | 5 | 6 |
| P047AN | M | 66 | Urban | Not at all | 11 | 2 |
| P048AN | F | 96 | Urban | Not at all | 5 | 6 |
| Legend for socio-professional categories:  1. Farmers.  2. Craftsmen, traders and business owners.  3. Managers and senior intellectual professions.  4. Intermediate professions.  5. Employees.  6. Labourers. | | | | | | |
